# Stroke Versus Seizure – Perfusion Computerized Tomography in a Patient with Aphasia

**DOI:** 10.5334/jbr-btr.880

**Published:** 2015-12-30

**Authors:** S. Dekeyzer, S. Vanden Bossche, V. Keereman, D. Hemelsoet, V. Van Driessche

**Affiliations:** 1 Department of Radiology and Medical Imaging, University Hospital Ghent (UZ Gent), Gent, Belgium; 2 Department of Neurology, University Hospital Ghent (UZ Gent), Gent, Belgium

**Keywords:** Perfusion CT, Postical aphasia, Seizure, Stroke, Stroke mimicry

## Abstract

Both stroke and seizures have varied clinical presentations and their differentiation in the acute setting is not always straightforward. We present the case of a patient who presented at the emergency room with acute onset aphasia. Clinically acute ischemic stroke was suspected. Perfusion CT was performed and demonstrated cortical hypervascularity in the left partietotemporal region. Additional MRI and EEG were performed and a final diagnosis of postictal aphasia was made. This case illustrates that perfusion CT is not only a useful tool for acute stroke management, but can also aid in the detection of seizures in patients presenting with stroke-like symptoms.

## Introduction

Stroke is a clinical diagnosis characterized by the acute onset of focal neurological symptoms. When patients present with acute neurologic deficits that can be attributed to a recognizable vascular territory the diagnosis is often straightforward. Differential diagnostic problems may arise however, because there are several stroke subtypes – not all of which are easily recognizable clinically – and because certain non-vascular medical conditions may simulate a stroke syndrome – a condition coined *stroke mimicry*.

It is estimated that up to one-fifth of cases identified as *brain attacks* are, in fact, due to such stroke-mimicking conditions [1]. In a large study with 512 patients who presented with acute neurologic deficits and received thrombolytic therapy, 21% were ultimately proven not to have suffered acute arterial ischemic strokes [2]. Stroke-mimicking conditions include hemiplegic migraine, infectious and metabolic disorders, intracranial tumours, nonconvulsive status epilepticus, Todd’s paresis, venous sinus thrombosis, and conversion disorder.

The differentiation between acute arterial ischemic stroke and stroke-mimicking conditions has important implications for patient treatment and management. Currently the standard treatment for acute ischemic stroke is the intravenous (IV) administration of recombinant tissue plasminogen within 3–4.5 hours from symptom onset. The risk of symptomatic intracranial haemorrhage following intravenous (IV) thrombolysis in patients with stroke mimickers is 1% [3]. Although this risk is low compared to the 7.9% risk in patients with acute ischemic stroke, it is not negligible. When possible, a correct diagnosis should be made to avoid the administration of IV thrombolysis and possible related complications in patients with stroke-mimicking conditions [3].

Non-contrast enhanced computed tomography (NECT) of the brain is currently the mainstay imaging study in patients suspected of suffering an acute arterial ischemic stroke, and its main role is to exclude intracranial haemorrhage. Unfortunately, NECT has a low sensitivity for the detection of early parenchymal changes in acute ischemic stroke [1]. Perfusion CT is capable of detecting regional differences in blood flow and is increasingly being used as an adjunct to NECT in the evaluation of stroke patients. Perfusion CT increases the diagnostic accuracy of acute ischemic stroke four-fold in non-expert readers compared to NECT – and may allow the identification of ischemic penumbra and infarct core – making the technique a potentially helpful tool in the selection of revascularization candidates for intravenous thrombolysis or endovascular procedures [4].

Perfusion CT can also help in detecting non-convulsive status epilepticus or postictal Todd’s phenomena and its utility has been proven in several studies and case reports [5,6,7,8,9,10,11,12]. In this paper we demonstrate the utility of perfusion CT in differentiating acute ischemic stroke from seizure by presenting the case of a patient with isolated postictal aphasia, followed by a brief review of the literature.

## Case Presentation

**Figure 1:**
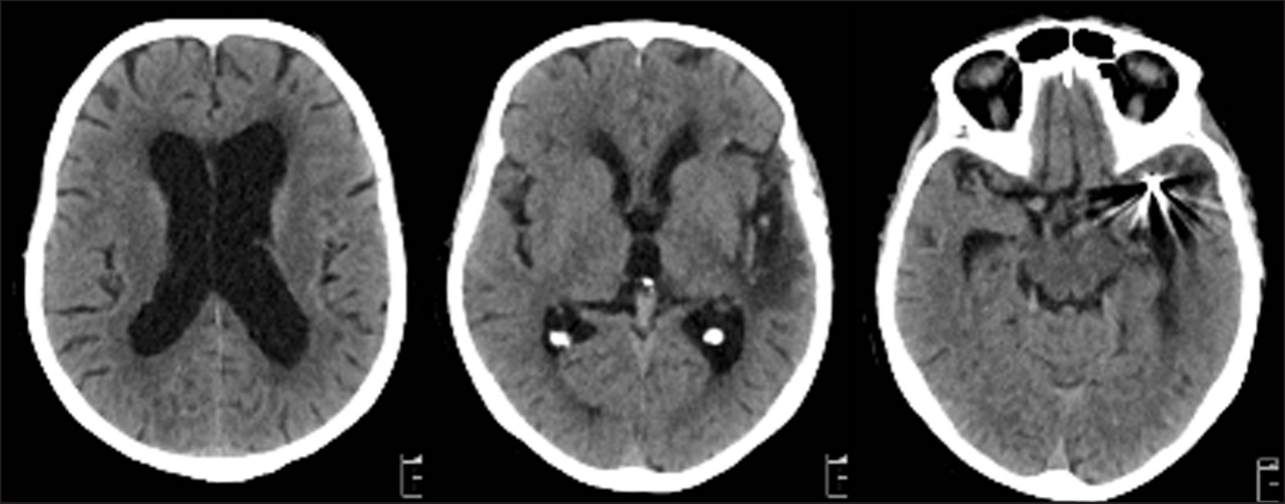
Unenhanced CT of the brain showed no abnormalities except known gliotic changes in the left temporo-insular region from an old haemorrhage and streaking artefacts from coiling material at the level of the left MCA-bifurcation.

**Figure 2:**
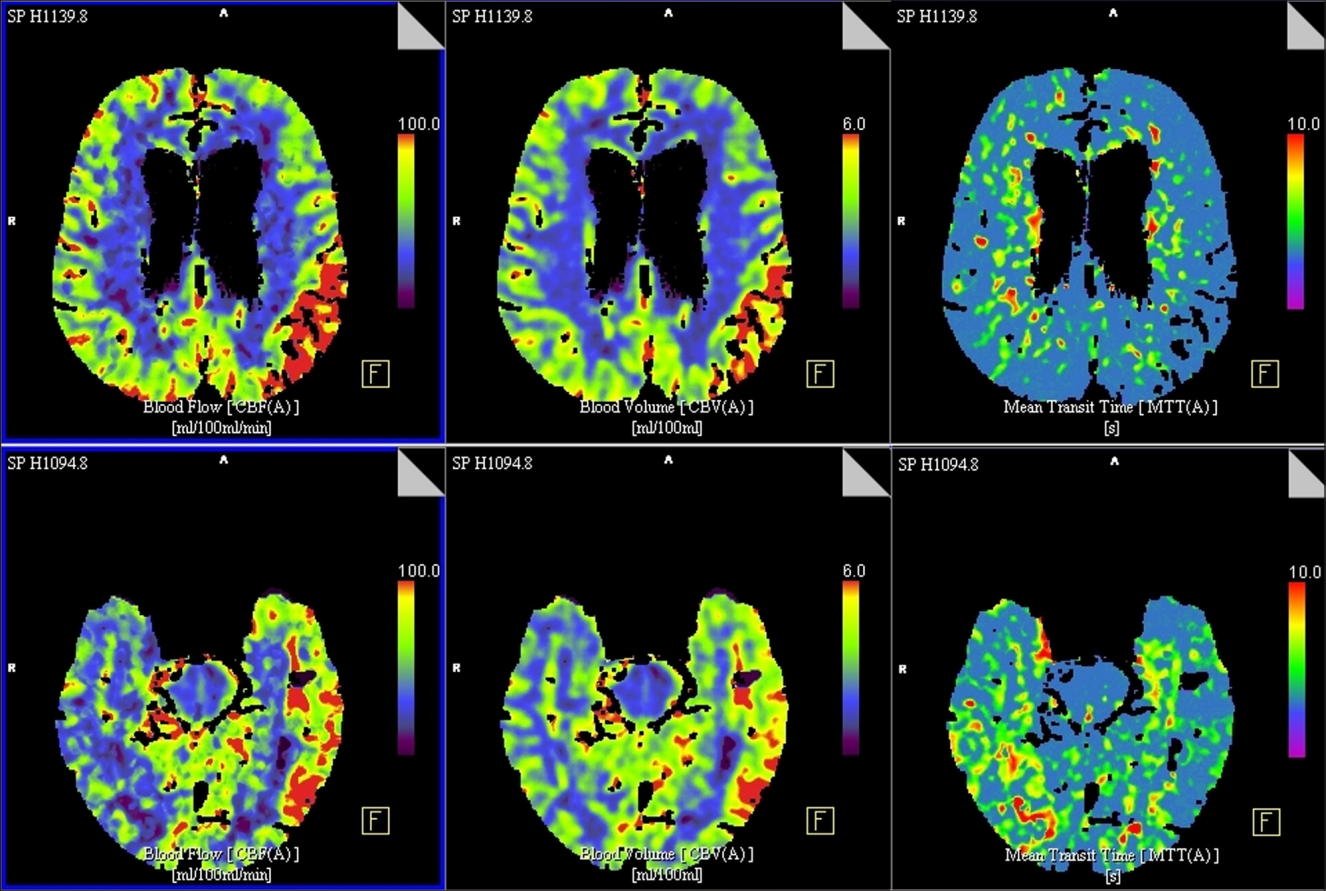
Top (**a**) and bottom (**b**) slices of brain perfusion CT shows extensive perfusion alterations in the entire left parietotemporal region, consisting of a normal to slightly diminished MTT (right column), and an increased CBF (left column) and CBV (middle column).

**Figure 3:**
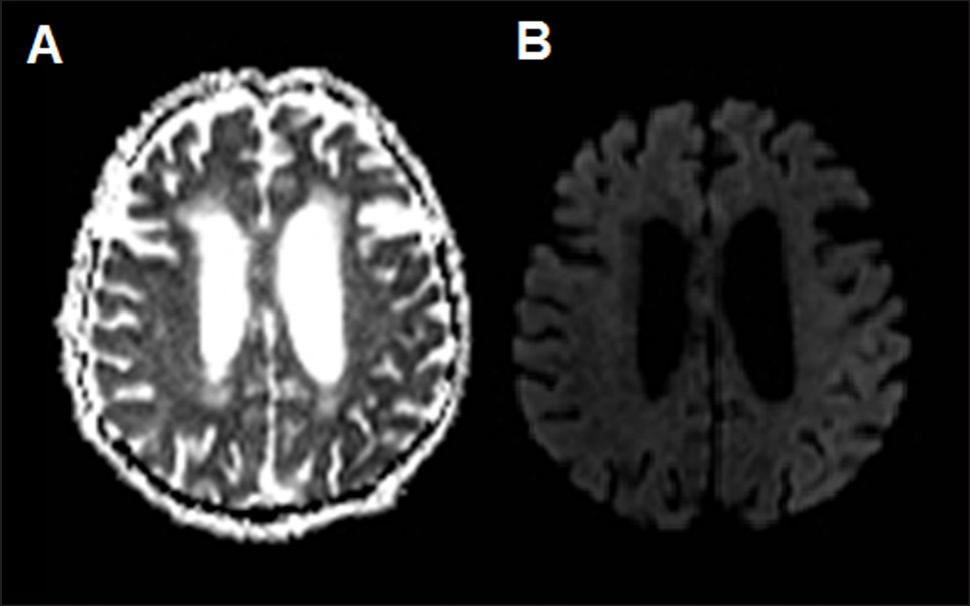
ADC (**a**) and b1000 DWI (**b**) show subtle diffusion restriction in the cortex of the left parietotemporal lobe, corresponding with the hyperperfused region observed on perfusion-CT.

An 80-year-old woman with a history of subarachnoid haemorrhage was brought to the emergency room because of speech problems. The patient was found in the morning by her relatives with severe speech comprehension and production difficulties. The exact time of symptom onset was unclear. Clinical examination at the ER revealed a sensory aphasia without other neurologic deficits. Stroke was suspected and the patient underwent a brain CT with an optimized stroke protocol consisting of an unenhanced CT, perfusion CT, and a CT angiography. Unenhanced CT showed no abnormalities, apart from already known and unchanged chronic hypodense gliotic changes in the left temporo-insular region (Figure 1). Perfusion CT showed a normal to slightly diminished mean transit time (MTT), a diminished time to drain (TTD) and a clearly increased cerebral blood flow (CBF) and cerebral blood volume (CBV) in the entire left parietotemporal region (Figure 2). No intra-arterial clots were seen on CT angiography. Urgent brain magnetic resonance imaging (MRI) was performed. Diffusion weighted images (DWI) showed subtle diffusion restriction in the left parietotemporal region, corresponding with the area of hyperperfusion on CT, without accompanying signal alterations on FLAIR (Figure 3). Based on the clinical presentation and imaging findings, a nonconvulsive status epilepticus or postictal Todd’s paresis was suspected. An Electroencephalography (EEG) was performed within two hours of the MRI. Only interictal epileptiform changes were seen with sharp theta waves and spike waves in the left temporal region. These EEG findings were not compatible with an ictal state – seizure or status epilepticus – as the discharges were neither rhythmic nor continuous. Therefore, a diagnosis of postictal sensory aphasia was made, presumably after a focal seizure involving Wernicke’s area. An antiepileptic treatment with levetiracetam was started. There was gradual improvement of the patient’s speech difficulties over three days and she was discharged home after six days without residual symptoms.

## Discussion

In the postictal phase transient focal neurologic deficits may appear lasting hours to days following focal onset seizure. They are described in up to 13.4% of seizure patients [13]. The classical presentation of Todd’s paresis is transient weakness of an arm, leg or hand following focal seizure activity within that limb. However, when seizures affect other areas than the motor cortex, other transient neurologic changes can be seen, such as sensory deficits, vision changes, neglect or aphasia. Clinically it can be difficult to distinguish postictal phenomena from acute ischemic stroke, especially when a (hetero-) anamnesis of the inciting seizure is lacking or when the seizure does not present with positive symptoms, as is often the case in seizures involving language areas.

In patients with suspected stroke, NECT is often the first imaging study to be performed. Unfortunately, NECT is not a sensitive radiologic tool in detecting stroke or in differentiating stroke from seizure. Perfusion CT is increasingly being used in the workup of patients with presumed ischemic stroke. Relative MTT, relative CBF and absolute CBV are CT perfusion parameters that help distinguish areas of infarct from potentially salvageable penumbra. Strokes typically present with a hypoperfusion pattern corresponding to a specific vascular territory and consisting of a relative increase in MTT and decrease in CBF. Absolute CBV values in stroke vary and are used to distinguish penumbra – (unchanged or increased CBV) from infarction – (decreased CBV) [14].

Several studies have systematically examined the changes on perfusion CT in seizure patients [5,6,7,8], and the utility of perfusion CT in differentiating stroke from seizure has been described in a number of several reports [9,10,11,12]. When discussing the spectrum of abnormalities on perfusion CT in seizure patients, a distinction has to be made between patients in status epilepticus and patients in the postictal phase.

The most common finding in patients with status epilepticus is cortical hyperperfusion, which can be seen in 78%–100% of patients and is characterized by a decrease in MTT and an increase in CBF and CBV [5,6,8]. Hyperperfusion in status epilepticus is generally limited to the cortex, does not involve the subcortical white matter and does not respect traditional vascular territories [8].

In postictal phenomena changes on perfusion CT are seen in only 30–37% of patients [7,8]. Imaging within two hours after seizure symptom termination is significantly associated with having an abnormal perfusion CT [7]. The most common abnormality on perfusion CT is focal hypoperfusion, characterized by prolonged MTT and decreased CBV and CBF, with involvement of both cortical and subcortical areas [7,8]. Though this pattern can resemble that of acute ischemic stroke, quantitative analysis of the perfusion parameters allows differentiation between these two entities. The focal relative CBF change, in the postictal state, accounts for an asymmetry index of 21%, compared to 80% for the core of an infarction and minimally, 34% for penumbra in acute ischemic stroke [8]. Furthermore, contrary to ischemic stroke, hypoperfusion due to postictal phenomena does not respect traditional vascular boundaries.

Hyperperfusion in status epilepticus is caused by the elevation of the local cerebral metabolic rate for oxygen and glucose, resulting in an increase in blood supply through neurovascular coupling [15]. In a similar way, postictal hypoperfusion is assumed to be the result of the normal metabolic coupling of perfusion to neuronal activity. In a state of postictal exhaustion or inhibition, this will result in regional reduction of CBF and CBV. Both CBF and CBV recover with a rebound in neuronal activity [11].

In our patient, a final diagnosis of postictal aphasia was made based on EEG findings which were not compatible with an ictal state. However, hyperperfusion was observed on perfusion CT, which contrasts with what has been described in most patients in the postictal state [7]. Only a limited number of cases can be found on postictal patients with hyperperfusion on perfusion CT and a convincing explanation for this finding is currently lacking [7,9,10]. One explanation is that our patient was still or temporarily suffering from seizure activity at the time of imaging. An alternative explanation is that prolonged and increased neural activity due to seizures results in the accumulation of products of anaerobic glycolysis in the neural tissues, which in turn, results in postictal enhancement of blood flow [16].

## Conclusion

This report illustrates that perfusion CT can play an important role in differentiating acute stroke from status epilepticus or postictal neurological deficits in the emergency room setting. Changes on perfusion CT are seen in most patients with status epilepticus and typically consist of cortical hyperperfusion. In postictal phenomena only a third of patients have abnormalities on perfusion CT and the chance of detecting abnormalities is significantly greater when imaging is performed within two hours of seizure termination. When perfusion CT alterations are present in postictal patients, they typically consist of cortical and subcortical hypoperfusion. Hyperperfusion has been described in a minority of patients with postictal paresis, but this might actually reflect ongoing seizure activity at the time of imaging.

## Competing Interests

The authors declare that they have no competing interests.
